# Development and Characterization of a Wound-Healing System Based on a Marine Biopolymer

**DOI:** 10.3390/gels11110881

**Published:** 2025-11-03

**Authors:** Catalina Natalia Cheaburu Yilmaz, Melisa Sirin Yildirim, Defne Govem, Hulya Ayar Kayali, Onur Yilmaz

**Affiliations:** 1 Biochemistry Division, Department of Chemistry, Faculty of Science, Dokuz Eylül University, 35390 Buca, Izmir, Türkiye; melisa58sirin@gmail.com (M.S.Y.); hulya.kayali@deu.edu.tr (H.A.K.); 2 Dokuz Eylül Üniversitesi İzmir Uluslararası Biyotıp ve Genom Enstitüsü; 35340 Balçova, Izmir, Türkiye; defne.govem@ibg.edu.tr; 3 İzmir Biyotıp ve Genom Merkezi, Dokuz Eylül Üniversitesi Sağlık Yerleşkesi, 35340 Balçova, Izmir, Türkiye; 4 Leather Engineering Department, Faculty of Engineering, Ege University, 35100 Bornova, Izmir, Türkiye; onur.yilmaz@ege.edu.tr; 5 Academichem Kimya ARGE San. Tic. Ltd. Şti, Ege University Technology Development Zone, Erzene Mah., Ankara Cad. No:172/67, 35100 Bornova, Izmir, Türkiye

**Keywords:** marine algae, Ulvan polysaccharide, boric acid, biopolymer modification, wound healing

## Abstract

Marine algae are a sustainable and eco-friendly resource, growing rapidly without freshwater or arable land while aiding carbon sequestration. Their extract is rich in biodegradable polysaccharides like alginate, fucoidan, carrageenan, agar, and Ulvan which can be used further in wound healing thanks to their unique characteristics such as ensuring moisture balance and tissue regeneration by forming biocompatible hydrogels with antimicrobial, anti-inflammatory, and antioxidant properties, key requirements in wound healing. The present study explored the utilization of local grown marine algae (i.e., Aegean seashores from Türkiye) and transforming the waste into useful end-products for dermatocosmetics and healing systems. The extracted polyssacharide, e.g., Ulvan which was characterized by means of FT-IR spectroscopy, DSC, and antioxidant activity, was included inside a semi-solid formulation and combined with other polysaccharides from other natural sources such a chitosan, alginate, and hyaluronic acid to form bioactive hydrogels with wound closure activity. The formulated hydrogels exhibited significant swelling capacity, antioxidant activity, and the selected optimal formulation exhibited enhanced wound closure rates in vitro, demonstrating potential for wound-healing applications.

## 1. Introduction

Chronic and non-healing wounds represent a major clinical challenge due to persistent inflammation, high risk of infection, and delayed tissue regeneration. Effective wound management strategies aim not only to protect against microbial contamination but also to maintain a moist environment, promote tissue repair, and prevent oxidative damage at the wound site [1,2]. Advanced wound dressings that combine these functions are therefore essential for improving therapeutic outcomes.

Hydrogels have gained prominence as advanced wound dressings owing to their ability to retain moisture, provide gas exchange, absorb exudates, and deliver bioactive compounds directly to the wound site [3]. Their structural similarity to the extracellular matrix, biocompatibility, and tunable properties make them suitable for supporting cell migration and tissue repair. Various natural and synthetic polymers, such as chitosan, alginate, hyaluronic acid, and polyvinyl alcohol (PVA), are frequently employed in hydrogel formulations to enhance their physicochemical and biological performance [4,5,6]. However, the search for alternative, sustainable biopolymers remains an important research priority.

Marine algae offer a renewable and eco-friendly source of bioactive polysaccharides. Unlike terrestrial resources, algae grow rapidly without requiring freshwater or arable land and contribute to carbon sequestration [7]. Among marine-derived polysaccharides, Ulvan, extracted from green macroalgae of the *Ulva* sp., has recently attracted interest for biomedical applications. Structurally, Ulvan is a complex sulfated polysaccharide composed primarily of rhamnose, glucuronic acid, iduronic acid, and xylose, with sulfate groups conferring distinctive physicochemical and biological activities [8]. Ulvan has demonstrated antioxidant, antimicrobial, anticoagulant, and immunomodulatory properties, positioning it as a promising candidate for tissue engineering and wound-healing applications [9,10,11]. However, its structure–activity relationship and the absence of standardized characterization protocols remain important limitations. Achieving a deeper understanding of how specific structural features—such as molecular weight, degree of sulfation, branching, and blending with other bio/polymers—influence its biological activity is crucial. Such knowledge would enable greater standardization and customization of Ulvan-based materials for personalized wound care approaches.

Blending Ulvan with other natural polymers may further enhance its biomedical potential. Specifically, chitosan may improve antimicrobial and hemostatic effects, hyaluronic acid promotes tissue hydration and cell migration, and alginate supports gelation and mechanical stability [12,13,14]. Such hybrid hydrogels could generate synergistic effects particularly beneficial for wound repair. Although several studies have described Ulvan-based hydrogels for biomedical applications, important gaps remain. Limitations such as insufficient mechanical robustness and excessive swelling after hydration restrict their suitability for durable topical applications. These issues can be addressed by optimizing polymer combinations and composition. Another strategy involves incorporating polyvinyl alcohol (PVA) into biopolymeric matrices, leveraging its ability to form hydrogen bonds via the freeze–thaw process, a simple and environmentally friendly crosslinking method.

The present study followed a stepwise screening approach. Initially, seven Ulvan-based hybrid hydrogels (UP1–UP7) were prepared by blending Ulvan with different natural polymers (chitosan, alginate, hyaluronic acid) and crosslinked with PVA to evaluate compositional effects. The primary objective was to identify the most suitable Ulvan-containing matrix for developing a multifunctional wound-healing system, focusing on compositions with the greatest potential for biomedical applications.

In this context, the Ulvan-based hybrid hydrogels were systematically evaluated for their physicochemical properties, swelling behavior, thermal stability, and antioxidant activity to assess their potential as topical wound-healing materials. Ulvan was selected as the primary functional component not only for its bioactive properties but also because it is abundantly available along the Aegean seashores as a waste biomass; its valorization into a high-value biomedical material may also help mitigate regional environmental pollution. Building on this systematic evaluation, a semi-solid formulation incorporating Ulvan-based polymer matrices together with boric acid, zinc oxide, and *Boswellia serrata* (frankincense) extract was designed to enhance antimicrobial, antioxidant, and anti-inflammatory functions. The central hypothesis is that this combination of marine-derived polysaccharides and supplementary bioactive agents will synergistically promote wound repair by providing both a protective matrix and active modulation of the wound microenvironment.

## 2. Results and Discussion

### 2.1. Structure Identity and the Extraction Efficiency of Ulvan

**Figure 1:**
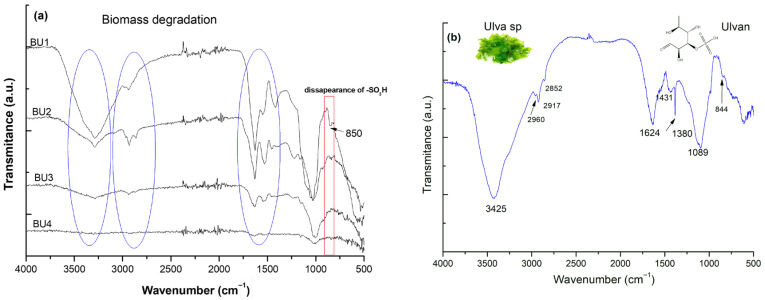
FT-IR spectra of *Ulva* sp. biomass after repeated extraction cycles (**a**) and the extracted and purified Ulvan polysaccharide (**b**).

The purpose of the extraction of Ulvan sp. sampled from the Aegean seashore was to obtain the sulphated polysaccharide, i.e., Ulvan. By repeated extraction cycles the degradation of the biomass (hereafter BU1-4) was followed up and the structure of Ulvan was confirmed by analyzing the FT-IR spectra. Figure 1a describes the FT-IR spectra of the biomass with an increasing number of cycles and Figure 1b reveals the spectroscopic characteristics of the extracted Ulvan.

The average extraction efficiency of Ulvan from *Ulva* sp. was found to be about 13 ± 4.3% [15], up to the specific type of *Ulva* species. For blade species (*U. papenfussii*), the extraction efficiency was reported as being 16% while for filamentous species, the crude extraction efficiency was about 10% [16] and Tran et al. [15] extracted Ulvan from *U. papenfussii* with an extraction efficiency of 14.8% based on the dry weight of seaweed. Within the present study, the average extraction efficiency, based on the dry weight of the *Ulva* sp. was determined within a range of 13–20%.

FT-IR spectra of the Ulvan extracts displayed characteristic absorption bands associated with polysaccharides, particularly signals corresponding to carboxyl and sulfate groups (Figure 1b). The overall spectral profiles were consistent with those previously reported for sulfated polysaccharides derived from different *Ulva* sp. [17,18,19,20,21].

A broad and intense band observed at 3400 cm^−1^ was assigned to O–H stretching vibrations, reflecting intra- and intermolecular hydrogen bonding within the polysaccharide matrix. A weaker shoulder around 2900 cm^−1^ indicated C–H stretching of aliphatic groups, typical for sugar residues. The strong absorption near 1089 cm^−1^ corresponded to C–O stretching of the rhamnose backbone. Sulfation of the polymer was evidenced by peaks in the 820–856 cm^−1^ range, assigned to C–O–S vibrations of axial sulfate groups, and by a shoulder around 1225–1226 cm^−1^, characteristic of S=O stretching of sulfate esters. Similar spectral features have been described for Ulvan extracted from *U. rigida* [22], *U. pertusa* [17], *U. lactuca* [18], and *U. clathrata* [20].

**Figure 2:**
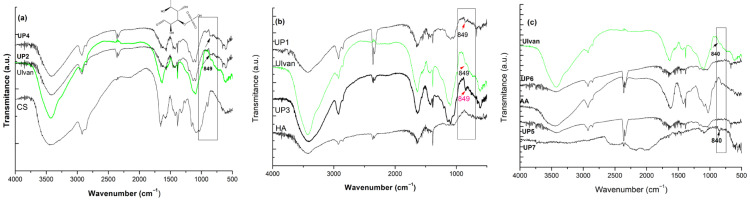
FT-IR spectra of Ulvan-based hydrogels prepared with chitosan (**a**), hyaluronic acid (**b**), and alginate (**c**).

Combining Ulvan with different biopolymers (i.e., chitosan- UP2 and UP4, alginate-UP5-6, UP7, and hyaluronic acid-UP1 and UP3) produced hydrogels which resembled the spectral characteristics of both combined biopolymers. Their structural characteristics are presented within Figure 2a–c.

More specifically, the characteristic bands of chitosan, hyaluronic acid, and alginate were preserved, while Ulvan-associated peaks were present as well, indicating a successful blending and crosslinking.

As seen within Figure 2, the FT-IR spectra of the hybrid hydrogels showed the characteristic bands of chitosan, hyaluronic acid, and alginate alongside those of Ulvan, confirming the successful incorporation of all components into a single network. Minor shifts and broadening in the O–H and C–O stretching regions from 3400 to 1620 cm^−1^ suggested the presence of hydrogen bonding and electrostatic interactions between the polymers, which indicate molecular compatibility rather than simple physical mixing. These interactions are expected to enhance the stability of the hydrogel structure, support its water retention capacity, and contribute to the functional performance required for wound-healing applications. Additionally, the peak corresponding to the sulfate groups of Ulvan from approximatively 840 cm^−1^ was determined as well within the spectra of the newly formed blends/hydrogels.

### 2.2. Thermal Properties

**Figure 3:**
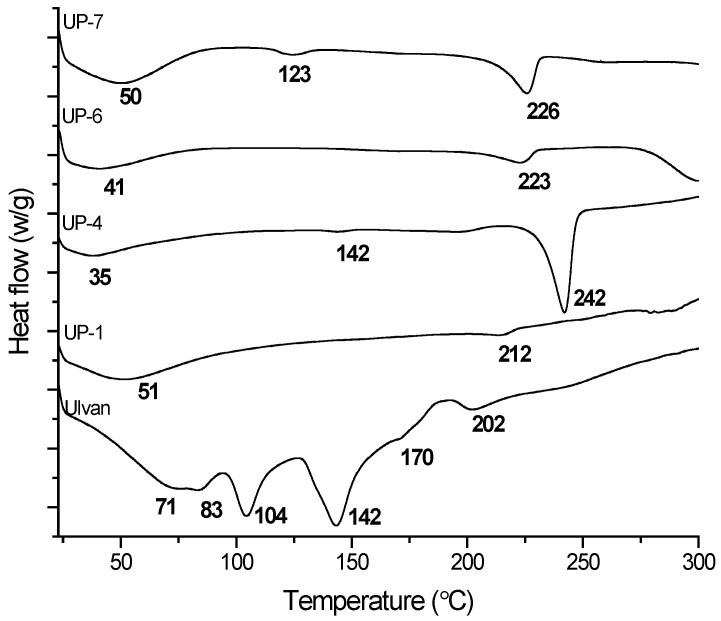
DSC thermograms of extracted Ulvan and its hydrogels with chitosan (UP2), hyaluronic acid (UP4), alginate (UP6), and PVA (UP7).

Differential scanning calorimetry (DSC) was used to evaluate the thermal behavior of Ulvan and its blends/hydrogels. Three main endothermic regions were observed (Figure 3). The peak at 118.8 °C, with an enthalpy change (ΔH) of 12.67 J/g, was consistent with reported values for *U. fasciata*, Ulvan (118.54 °C) and was attributed to the evaporation of physiosorbed water from the polysaccharide network. Similar low-temperature endothermic events have been reported for Ulvan/alginate systems, where a broad transition between ~35 °C and 161 °C was assigned to moisture loss, supporting our interpretation of this transition [23]. Additional observed peaks between 148.1 °C and 209.3 °C corresponded to successive degradation steps of the Ulvan backbone, consistent with the findings of Sulastri et al. [24]. The DSC profiles of the Ulvan-based hydrogels (UP1, UP4, UP6, UP7) showed variations in both peak position and enthalpy values depending on the co-biopolymer, indicating hydrogen bonding and electrostatic interactions within the blends. Comparable behavior was reported by Mujalli et al. in the case of chitosan/PEO composites, where degradation peaks shifted between ~150 and 200 °C depending on the composition [25]. In the case of alginate/PVA-based films, structural stability was maintained up to 240–260 °C before major breakdown as reported by Musa et al. [26], suggesting that the incorporation of additional stabilizers or optimized crosslinking strategies could further improve the thermal resistance of Ulvan-based hydrogels. Overall, these results confirm that combining Ulvan with natural polymers such as alginate, chitosan, and hyaluronic acid enhanced the thermal profile and stability, these outcomes supporting its application as a robust wound-dressing material.

### 2.3. Swelling Ability

**Figure 4:**
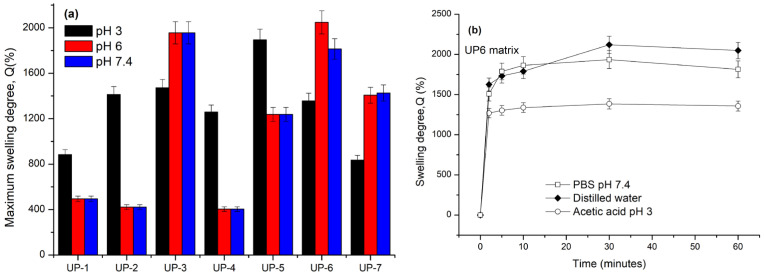
Maximum swelling capacity of the Ulvan-based hydrogels in different media (aqueous acetic acid pH3; twice-distilled water pH 6; and phosphate buffer solution pH7.2 at room temperature) (**a**) and the swelling profile of a selected hydrogel (UP 6) (**b**). Data are expressed as mean ± SD (*n* = 3) and the significance was determined by one-way ANOVA, *p* < 0.05.

The swelling behavior of Ulvan-based hydrogels is represented within Figure 4.

The swelling behavior of Ulvan-based hydrogels was strongly dependent on the surrounding medium. The highest swelling capacity was observed in phosphate-buffered saline (PBS, pH 7.2), indicating that physiological-like conditions favored water uptake and network expansion. Moderate swelling occurred in distilled water (pH 6), whereas acidic conditions (acetic acid, pH 3) led to reduced swelling, likely due to protonation of functional groups and tighter polymer packing. This pH-responsive behavior is advantageous for wound dressings, as local pH varies from acidic in infected sites to near-neutral during healing, where enhanced swelling facilitates exudate absorption and bioactive diffusion.

Among the tested hydrogels, UP6 exhibited the highest swelling ratio (482 ± 18%), closely followed by UP7 (465 ± 22%), with no statistically significant difference between the two (*p* > 0.05). These results confirm that UP7 displayed swelling behavior comparable to UP6, while maintaining a more uniform structure suitable for semi-solid formulation. The observed swelling values are in agreement with previously reported Ulvan-based systems, which typically range between 400% and 500% under similar conditions [27,28,29]. For instance, Ulvan–chitosan hydrogels have shown ~400% swelling, while alginate/PVA/Ulvan hybrids exhibited 130–190% after several hours in aqueous media [28].

Overall, the swelling performance of UP6 and UP7 suggests effective exudate management under physiological conditions, and further optimization of crosslink density or polymer blend ratios may enhance their functionality for advanced wound care applications.

### 2.4. Antioxidant Activity of the Ulvan-Based Hydrogels

**Figure 5:**
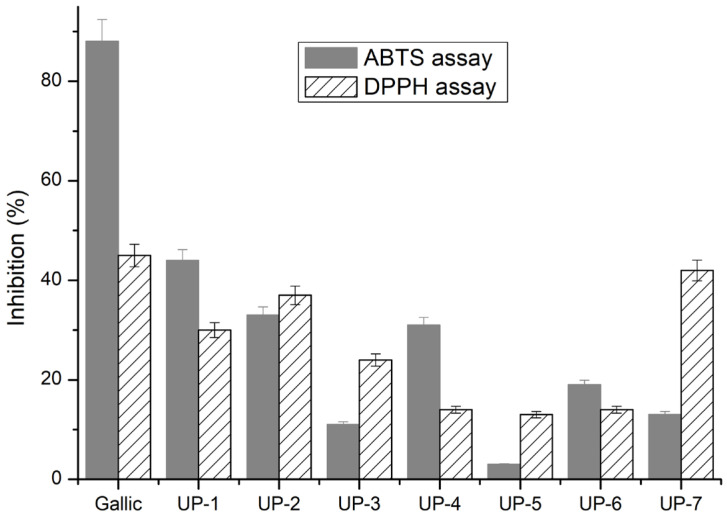
DPPH and ABTS radical scavenging activity of UP5–UP7 hydrogels. Data are presented as mean ± 5% (*n* = 3) with one-way ANOVA; *p* < 0.05 was considered significant.

Antioxidant activity was assessed by ABTS and DPPH assays and the inhibition of each Ulvan-based matrix was determined by using Equation (1) and the activity is represented within Figure 5 by comparison with the inhibition of gallic acid which was considered as positive standard.

As shown in Figure 5, the DPPH scavenging activity increased significantly with the Ulvan content of the formulation (*p* < 0.05, one-way ANOVA). UP7 exhibited the highest DPPH inhibition (42.1 ± 1.8%), which was significantly higher than that of UP6 (18.2 ± 1.1%) and UP5 (15.8 ± 0.9%) according to Tukey’s post hoc test (*p* < 0.05). A similar trend was observed for ABTS radical inhibition, where UP7 (40.3 ± 1.5%) outperformed UP6 (20.7 ± 1.2%) and UP5 (17.4 ± 1.0%) (*p* < 0.05). These results confirm that the PVA/Ulvan 80:20 composition (UP7) provides the most effective radical scavenging network among the tested formulations. This enhancement can be attributed to the higher relative content of Ulvan, which contains sulfate and hydroxyl groups capable of donating hydrogen atoms, as well as to the balanced PVA/Ulvan ratio (80:20) that preserves chain mobility and facilitates radical quenching. In contrast, UP5 and UP6 contain higher alginate proportions, leading to denser crosslinking and reduced accessibility of active sites. Therefore, the superior DPPH response of UP7 likely arises from its optimized polysaccharide–polymer network enabling effective diffusion of free radicals and stabilization of radical intermediates.

Hydrogels with higher Ulvan content, particularly UP6 and UP7, exhibited stronger antioxidant responses than lower-Ulvan formulations. After 30 min, ABTS radical scavenging activity reached about 28 ± 3% for UP6 and 33 ± 4% for UP7, while DPPH inhibition was 32 ± 3% and 39 ± 4%, respectively. The enhanced activity can be attributed to the presence of Ulvan’s sulfate and uronic acid groups, which act as hydrogen donors and radical stabilizers within the PVA-based network. These data confirm that increasing Ulvan loading improves the overall antioxidant potential of the hybrid hydrogels.

These results suggest that sulfate groups and uronic acids in Ulvan directly contributed to radical neutralization as also reported by other researchers [28,29,30].

The observed differences between ABTS and DPPH assays underscore the importance of employing multiple methods, as each probes distinct antioxidant mechanisms: ABTS primarily reflecting electron transfer and DPPH being more sensitive to hydrogen atom donation [31,32].

From a biomedical standpoint, the strong free radical scavenging ability of UP7 is highly advantageous, since excessive reactive oxygen species can delay chronic wound healing [33]. Compared to other marine polysaccharide-based hydrogels reported in the literature, UP6 demonstrated comparable or higher antioxidant potential, reinforcing Ulvan’s utility as a multifunctional biomaterial for wound care applications [30,31]. More particularly, Wang et al. [30] found, for Ulvan with calcium alginate, a scavenging activity between 35% and 60% up to the concentration of calcium ions which were shown to have a big contribution to the scavenging activity. UP7 exhibited the highest DPPH radical scavenging activity (≈42%), markedly exceeding UP6 (≈18%) and UP5 (≈16%). This enhancement can be attributed to the higher relative content of Ulvan, which contains sulfate and hydroxyl groups capable of donating hydrogen atoms, as well as to the balanced PVA/Ulvan ratio (80:20) that preserves chain mobility and facilitates radical quenching. In contrast, UP5 and UP6 contain higher alginate proportions, leading to denser crosslinking and reduced accessibility of active sites. Therefore, the superior DPPH response of UP7 likely arises from its optimized polysaccharide–polymer network enabling effective diffusion of free radicals and stabilization of radical intermediates.

A direct comparison between UP6 and UP7 (Table 1) have shown that although UP6 exhibits the highest swelling in PBS (~1700%), UP7 presents substantially higher antioxidant activity (ABTS ~40% and DPPH ~42% at 0.5 mg/mL) while retaining comparable swelling (~1400%). Moreover, UP7’s simpler PVA/Ulvan matrix (80:20) allowed more reproducible incorporation of boric acid, ZnO and *Boswellia serrata* (frankincense) actives into the semi-solid YY during mechanical mixing (reduced phase separation and cream-like rheology). Taken together, the antioxidant advantages, comparable swelling, and superior processability motivated the selection of UP7 as the basis for YY.

### 2.5. SEM Observations

**Figure 6:**
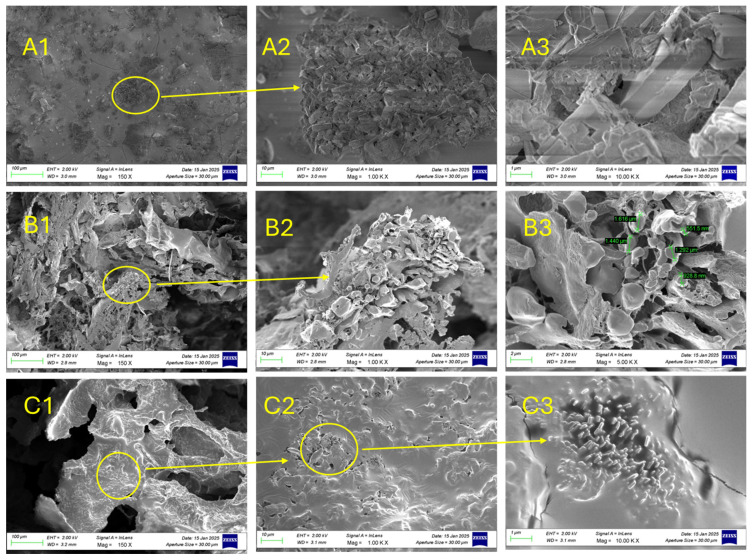
SEM observations for the Ulvan-based systems at different magnifications, i.e., 150X; 1,00 KX; and 10,00 KX: (**A1**–**A3**) purified Ulvan, (**B1**–**B3**) AA/PVA/Ulvan (UP6), and (**C1**–**C3**) lyophilized Ulvan/PVA (UP7).

Morphological analysis of the selected hydrogel formulation was conducted to investigate its internal microstructure and confirm whether its physical architecture supports the functional properties observed in swelling and antioxidant assays. The arrangement of pores, their size distribution, and interconnectivity are critical determinants of hydrogel performance, as they govern water retention, mechanical stability, and the diffusion of bioactive molecules. For wound-healing applications, a highly porous and interconnected network is desirable to promote exudate absorption, oxygen and nutrient transport, and cellular infiltration [34]. Therefore, analyzing the morphology of UP6 and UP7 as selected matrices due to high swelling degree and antioxidant activity, was essential to validate that the superior swelling profile is linked to an appropriate internal structure, ensuring that the formulation can effectively serve as a wound-dressing material. SEM observations for the Ulvan-based systems, e.g., purified Ulvan (Figure 6A1–A3), AA/PVA/Ulvan (UP6—Figure 6B1–B3) and lyophilized Ulvan/PVA (UP7—Figure 6C1–C3) at different magnifications were selected based on their performance and distinct morphological features (Figure 6).

As shown in Figure 6A1–C3, the selected hydrogels displayed a porous and interconnected morphology.

SEM micrographs revealed that purified Ulvan (Figure 6A1–A3) exhibited a flaky and irregular morphology, consistent with its polysaccharide structure. The UP6 sample (Figure 6B1–B3) exhibited larger, more irregular pores, while UP7 (Figure 6C1–C3) showed a denser, more uniform structure.

Such morphology is highly desirable for wound-healing applications, as it facilitates fluid absorption, oxygen diffusion, and cell infiltration. Lyophilization further enhanced pore visibility, indicating that processing method strongly influences microstructural features. The presence of homogeneous and interconnected pores in Ulvan-based blends suggested good compatibility between polymers and predicted favorable swelling and drug release behavior. These observations confirm that the microarchitecture of the hydrogels supports their intended biomedical function.

### 2.6. Rheological Behavior of the Prepared Semi-Solid Formulation

**Figure 7:**
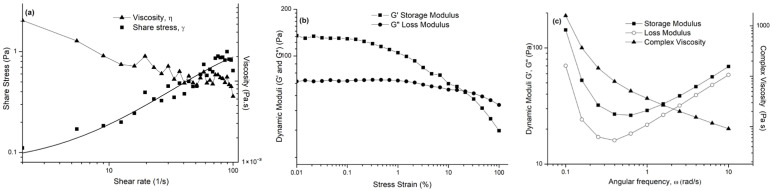
Rheological profile of the semi-solid formulation: (**a**) flow behavior; (**b**) amplitude sweep test; and (**c**) viscoelastic behavior (frequency sweep test).

In Figure 7a–c, the rheological profile of the prepared semi-solid formulation (YY) is represented.

The rheological profile of the semi-solid formulation (YY) demonstrated a characteristic non-Newtonian, pseudoplastic behavior, consistent with previously reported trends for semi-solid pharmaceutical and cosmetic systems [35]. As shown in Figure 7a, the viscosity (η) decreased markedly from approximately 850 Pa·s at 0.1 s^−1^ to 95 Pa·s at 100 s^−1^, confirming strong shear-thinning behavior. Such behavior is typical of polymeric or emulsion-based gels, where the internal structure resists flow at rest but aligns under shear, facilitating easy spreadability and ensuring physical stability during storage. The amplitude sweep test (Figure 7b) revealed that the storage modulus (G′) exceeded the loss modulus (G″) within the linear viscoelastic region (0.1–1% strain), indicating a predominantly elastic, solid-like network similar to those described for stable topical gels and creams [35,36,37]. The decline of both moduli beyond a critical strain of ~5% suggests disruption of the internal network, marking the yield point, which defines the onset of irreversible flow. In the frequency sweep test (Figure 7c), G′ remained greater than G″ throughout the frequency range (0.1–10 rad·s^−1^), confirming a dominant elastic response and well-organized microstructure. The complex viscosity (η*) decreased from approximately 600 Pa·s to 150 Pa·s with increasing frequency, further supporting the shear-thinning and viscoelastic behavior commonly reported for structured semi-solids. Collectively, these results align with the literature findings indicating that formulations exhibiting high G′ values, low frequency dependence, and shear-thinning flow profiles possess enhanced structural stability, resistance to phase separation, and desirable application properties such as smooth spreadability and ease of use [36,37].

### 2.7. Cytotoxicity Assessment

All biological experiments (cytotoxicity and scratch assay) were carried out on the final semi-solid YY formulation, while structural and physicochemical analyses correspond to its UP7 hydrogel base.

**Figure 8:**
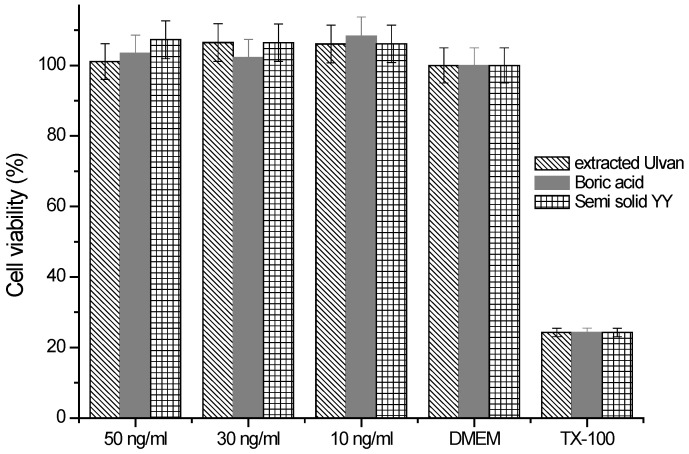
Cell viability assessment of pure Ulvan, boric acid, and the prepared semi-solid formulation from UP7 at different doses against the positive (DMEM) and negative (TX-100) controls. Data are expressed as mean ± 0.5 (*n* = 3); significance was determined by one-way ANOVA, *p* < 0.05.

The cytotoxicity evaluation (described in Figure 8) demonstrated that both pure Ulvan and the formulated hydrogel (YY) were well tolerated by fibroblasts, as cell viability remained comparable to the positive control (DMEM) across all tested doses, while TX-100 (negative control) confirmed assay sensitivity. These findings underline the biocompatibility of Ulvan-based systems, consistent with previous reports highlighting Ulvan’s low toxicity and potential for biomedical applications [29,38].

**Figure 9:**
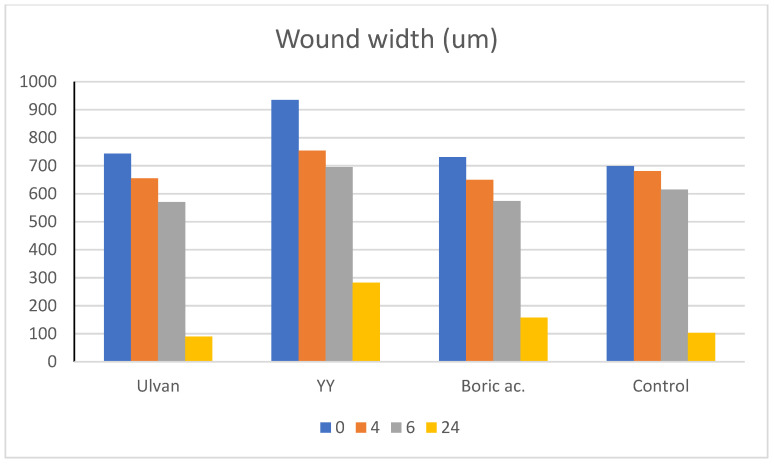
Wound width determination after administration of doses of boric acid, Ulvan, and formulated YY by scratch assay. Data are expressed as mean ± 0.5 (*n* = 3). Statistical analysis and plotting were performed using OriginPro 2023 (OriginPro, Version 8.1; Origin Lab Corp.: Northampton, MA, USA). Significance was determined by one-way ANOVA, *p* < 0.05.

The scratch assay (Figure 9, Table 2) provided further insight into the wound-healing potential of the tested samples. Ulvan alone enhanced migration rates compared to the untreated control (22.11 µm/h at 4 h vs. 4.59 µm/h in control), confirming its intrinsic bioactivity. This is in line with previous studies reporting that Ulvan and other sulfated polysaccharides promote fibroblast proliferation and migration through modulation of growth factor signaling and extracellular matrix interactions [39,40].

Most notably, the YY formulation induced the strongest effect in the early wound-healing phase, with migration rates of 45.18 µm/h (4 h) and 39.92 µm/h (6 h). This synergistic enhancement is attributable to the combination of Ulvan with boric acid and frankincense actives, which likely act on complementary pathways. Similar early stimulatory effects have been reported for boron-containing dressings, where boric acid accelerated wound closure and reduced microbial load. Frankincense-derived compounds, particularly *Boswellia serrata* (Frankincense) actives, have also been associated with anti-inflammatory activity and enhanced keratinocyte migration. Together, these components appear to confer rapid fibroblast motility, which is clinically important, the formulation possessing putative antimicrobial/antiseptic function that requires dedicated microbiological evaluation.

**Figure 10:**
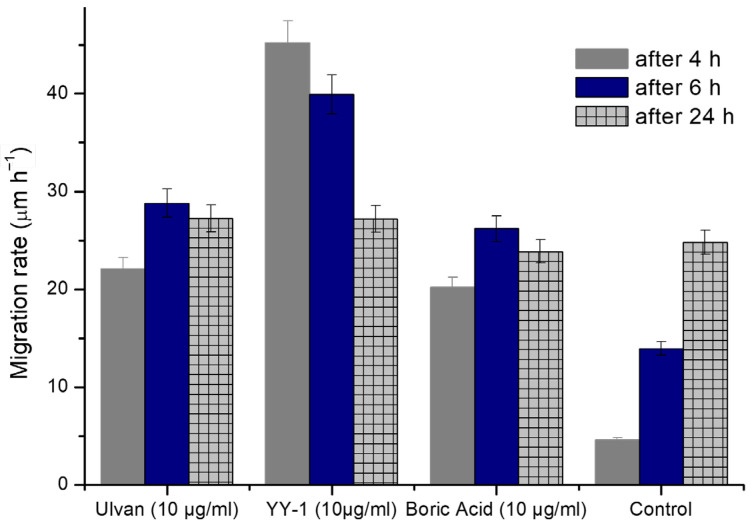
The migration rate of the treated cells with the studied matrices after 4, 6, and 24 h from the treatment with Ulvan, boric acid, and formulation YY. Data are expressed as mean ± 1 (*n* = 3). Significance was determined by one-way ANOVA, *p* < 0.05.

Interestingly, after 24 h, migration in YY-treated cells (27.19 µm/h) plateaued, becoming comparable to Ulvan (27.25 µm/h) and control (24.80 µm/h) (Figure 10). This suggests that YY primarily accelerates the early phases of wound closure rather than sustaining long-term migration. Such a profile may be beneficial in acute wound settings where rapid stabilization is critical, as opposed to chronic wounds that require prolonged stimulation.

The plateau observed after 24 h may result from the depletion or equilibrium of diffusible bioactive agents (boric acid, ZnO, and Boswellia extract) at the wound interface. These components likely promoted rapid early cell migration through antioxidant and signaling effects, but their influence diminished once cells reached near confluence. The slowdown could also reflect the natural transition from migration to proliferation phases in fibroblast cultures. Future studies will investigate these mechanisms through time-dependent release and live-cell migration analyses.

Boric acid treatment also improved migration relative to control (20.20–26.20 µm/h across the time course), though to a lesser degree than Ulvan or YY, confirming its supportive but not dominant role in fibroblast motility.

**Figure 11:**
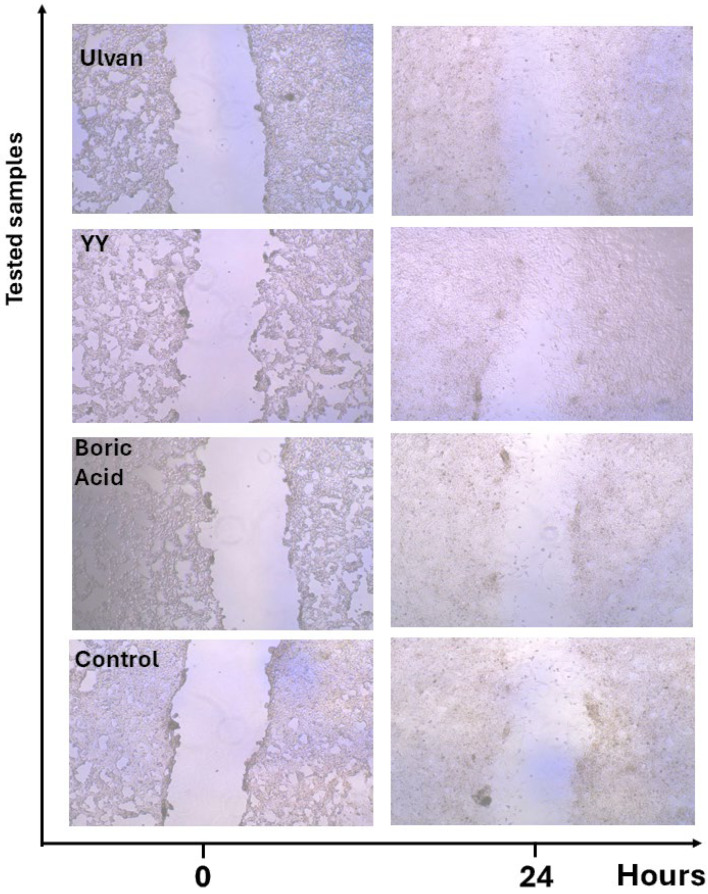
Microscopic snapshots of the wound closure activity after 24 h from the treatment of the cells with the tested samples.

Microscopy images (Figure 11) corroborated these quantitative data, showing narrower wound gaps in Ulvan and especially in YY-treated groups compared to untreated controls. Similar microscopic evidence of accelerated wound closure has been documented in Ulvan-based hydrogel dressings applied to in vitro and in vivo wound models [41].

Overall, these results highlighted the cytocompatibility of Ulvan-based hydrogels and demonstrated that the YY formulation accelerates fibroblast migration, particularly during the early healing window.

Literature comparisons further supported that combining Ulvan with boric acid and bioactive plant extracts can be a promising multifunctional strategy for wound management. Ulvan-based hydrogels crosslinked with boric acid have been shown to display enhanced swelling, antioxidant capacity, and antimicrobial activity compared to Ulvan alone, underscoring the synergistic effects of this combination [29]. In addition, boric acid-containing wound dressings have been clinically reported to stimulate epithelialization, granulation, fibroblast proliferation, and angiogenesis, thereby accelerating wound closure compared with conventional dressings [42]. The incorporation of bioactive plant extracts offers further functionality, for example, frankincense essential oil has been demonstrated to downregulate inflammatory cytokines, enhance collagen deposition, and significantly improve wound contraction [43,44,45]. Similarly, psyllium–frankincense hydrogels have been shown to possess superior antioxidant and antimicrobial properties while accelerating wound healing in animal models [45]. Taken together, these findings highlight that integrating Ulvan with boric acid and selected phytochemicals can yield multifunctional hydrogels capable of addressing the complex requirements of wound environments, including exudate absorption, oxidative stress mitigation, and tissue regeneration.

Although no antimicrobial assays were performed in this study, the inclusion of chitosan, boric acid, zinc oxide, and *Boswellia serrata* (Frankincense) extract is expected to confer antimicrobial potential to the formulation. These components are known to inhibit the growth of common wound pathogens such as *Staphylococcus aureus* and *Escherichia coli*. Future studies will aim to validate this anticipated antimicrobial effect through targeted microbiological testing.

## 3. Conclusions

The present study reported the preparation and characterization of various Ulvan-based hydrogels, highlighting their potential as multifunctional biomaterials. Ulvan was extracted from *Ulva* sp., a marine green alga from the Aegean considered unlikely to bloom and thus treated as waste biomass. The extracted Ulvan, a sulphated polysaccharide, is known for its biological properties and its potential for biomedical and pharmaceutical use. However, its structural variability and limited mechanical strength remain major drawbacks. Combination and crosslinking with other biopolymers, e.g., alginate, chitosan, hyaluronic acid, etc., lead to materials with higher mechanical stability, tunable swelling behavior, and functional bioactivity. Various hydrogels comprising chitosan, alginate, and hyaluronic acid with various compositions of Ulvan were prepared and characterized. The performed swelling studies demonstrated that the hydrogels possess tunable water uptake capacity, influenced by the composition and crosslinking density, which is critical for wound dressing and drug delivery applications. SEM analysis of selected samples (UP6 and UP7) revealed a highly porous and interconnected network, indicating favorable conditions for cell infiltration and nutrient transport. Thermal analysis via DSC confirmed the stability of Ulvan and its hydrogels, aligning with literature-reported thermal properties, suggesting that these hydrogels can maintain structural integrity under physiological conditions.

Moreover, the literature comparisons further support that combining Ulvan with other constituents such as boric acid and bioactive plant extracts provided a promising approach to enhance antimicrobial, antioxidant, and healing properties, thereby offering a multifunctional strategy for wound management. Overall, the findings indicate that Ulvan-based hydrogels were versatile, biocompatible, and potentially effective for biomedical applications, laying the groundwork for further in vivo studies and clinical translation.

## 4. Materials and Methods

### 4.1. Materials and Reagents

Poly (vinyl alcohol) (PVA, Mw 89,000–98,000, 99% hydrolyzed), alginic acid sodium salt (AA), chitosan of medium molecular weight (CS)-CAS 9012-76-4, SKU448877, hyaluronic acid (HA)-CAS 9004-61-9, SKU935166, Triton X-100 (TX-100, non-ionic surfactant used for gentle membrane permeabilization), boric acid (H_3_BO_3_), zinc oxide (ZnO) nanopowder, and phenoxyethanol (FE) were purchased from Sigma-Aldrich (St. Louis, MO, USA). Ulva sp. biomass was collected from the Aegean coast (Turkey) and used for Ulvan extraction as described. The *Boswellia serrata* (Frankincense) extract was commercially achieved as a botanical extract from a local pharmacy, an extract of Boswellia serrata 100% pure undiluted therapeutic grade.

All other chemicals were analytical grade and used without further purification. Ultrapure water was used in all preparations.

For the determination of antioxidant activity there were 1,1-diphenyl-2-picrylhydrazyl radical (DPPH), 2,2′-Azino-bis(3-ethylbenzothiazoline-6-sulfonic acid) diammonium salt (ABTS assay), methanol, ethanol, and gallic acid used as standard, all Sigma-Aldrich products.

For the cell culture studies, NIH-3T3 mouse embryo fibroblasts were maintained in Dulbecco’s Modified Eagle’s Medium (DMEM) supplemented with 10% fetal bovine serum (FBS), 100 IU/mL penicillin, and 100 μg/mL streptomycin, at 37 °C in a humidified atmosphere containing 5% CO_2_.

### 4.2. Extraction of Ulvan

For the extraction of Ulvan, 5 g washed and dried *Ulva* sp. was weighed and placed in a two-neck balloon equipped with magnetic stirrer and heater. A total of 100 mL of solution of 0.1 M HCL was used and the extraction was kept for 4 h at 90 °C with a mixing rate of 200 rpm. After each step, the biomass was filtered and a new portion of acid solution was added again. The extraction procedure was repeated for three cycles. The filtrate was collected after each filtration lyophilized and purified in ethanol. The remaining biomass after each extraction cycle was analyzed by means of FTİR and the degradation of the structure as Ulvan was extracted (BU1-3) was monitored. The extraction system was optimized considering the FT-IR results. After the extraction the collected filtrates were freeze-dried resulting in soft sponge-like materials. A further purification is preceded by dissolving the lyophilized materials in a small amount of twice-distilled water (about 20 mL) and purified again by precipitation in cold ethanol in a ratio of extract solution to cold ethanol of 1:1.5 *v*/*v*. The precipitates, appeared like white fine particles, were centrifuged at 4 °C, 6000 rpm for 15 min. The final Ulvan powder was obtained after lyophilization of the precipitate. The final extracted Ulvan was collected as a light beige powder. The extraction efficiency (%) was calculated as the ratio between the dry weight of purified Ulvan obtained after ethanol precipitation and the initial dry weight of *Ulva* sp. powder used for extraction. The yield ranged between 13% and 20%, depending on seasonal variations in the polysaccharide content of the collected biomass and minor differences in extraction efficiency among batches.

### 4.3. Preparation of Hydrogels

Table 3 summarizes the prepared hydrogels and formulation by using the extracted Ulvan. The polymer composition ratios (e.g., PVA/Ulvan/alginate = 50:30:20 for UP1) were selected based on a combination of preliminary optimization trials and previous experience with physically crosslinked hydrogel systems. The inclusion of PVA as a synthetic polymeric component was intended to enhance the gel strength, elasticity, and textural stability of the biopolymeric matrix, addressing some of the inherent weaknesses of polysaccharide-based gels. Previous studies on similar PVA-based hydrogels have shown that both the PVA concentration and the number of freeze–thaw cycles markedly influence properties such as swelling degree, mechanical behavior, and release kinetics of incorporated bioactives [46]. Therefore, a moderate PVA content (30 wt%) and two freeze–thaw cycles were chosen to achieve sufficient crosslinking and stability while maintaining flexibility and biopolymer integrity suitable for semi-solid formulation development.

### 4.4. Characterization Methods

The study design followed a stepwise screening approach. Initially, seven Ulvan-based hybrid hydrogels (UP1–UP7) were prepared by blending Ulvan with different natural polymers (chitosan, alginate, hyaluronic acid) and a synthetic polymer (PVA) to evaluate compositional effects. The aim was to identify the most suitable Ulvan-containing matrix for a multifunctional wound-healing system focusing on the evaluation of the most relevant compositions for biomedical application.

#### 4.4.1. Fourier Transform Infrared Spectroscopy (FT-IR) Analysis

The structures of the extracted Ulvan and its conjugated compounds with the other biopolymers (i.e., hyaluronic acid, chitosan, alginic acid) were confirmed by FT-IR spectra which were recorded with a Perkin-Elmer Spectrum-100 ATR-FT-IR instrument (Midland, ON, Canada). The analyses were performed on the dried biomass and lyophilized biopolymeric samples. The spectrum was obtained after 5 scans between 4000 and 600 cm^−1^, using reflection on a diamond crystal with an angle of 45° and a resolution of 4 cm^−1^.

#### 4.4.2. Swelling Tests

To determine the swelling capacity of the hydrogels in different pH solutions, acetic acid (pH = 3), water (pH = 5.4), and PBS solution (pH = 7.4) were used. The hydrogels were immersed in acetic acid, water, and PBS solution (25 mL) at pH 3, 5.6, and 7.2 (room temperature), respectively. When the maximum degree of absorption was reached, the sample was slowly removed from the solutions and the excess solutions on the surface of the sample were dried with filter paper and then the sample was weighed again. The degree of swelling was calculated with the following equation:

% Degree of swelling = (W_s_ − W_d_)/W_d_ × 100 (1)

where W_s_ is the weight of the blown film at a given time and W_d_ is the weight of the initial film (dried film).

#### 4.4.3. Differential Scanning Calorimetry (DSC)

The observed phase-change analyses, such as glass transition temperatures DSC analysis, were performed on dried substances (approximatively 5 mg) using a Perkin Elmer Diamond DSC instrument at a heating rate of 10 °C/min under a N_2_ atmosphere from 20 to 250 °C.

#### 4.4.4. Scanning Electron Microscopy (SEM)

The scanning electron microscope (SEM)-based images of the cross-section of the lyophilized selected samples were captured using a Carl Zeiss (Jena, Germany) 300 Sigma VP model equipped with Gemini Optical Technology. The magnification is given in the figures.

#### 4.4.5. Antioxidant Activity—DPPH and ABTS Analysis

Natural antioxidants are generally preferred over synthetic ones due to their safety and bioactivity. Accordingly, various methods have been developed to evaluate antioxidant potential, with the 1,1-diphenyl-2-picrylhydrazyl (DPPH) radical scavenging assay being among the most widely used. Common synthetic antioxidants include butylated hydroxytoluene (BHT), propyl gallate (PG), butylated hydroxyanisole (BHA), and tert-butylhydroquinone (TBHQ) [32,47].

For the DPPH assay, antioxidant activity was expressed as the percentage of radical inhibition, calculated using Equation (2):

(2) $$\%Inhibition=\frac{{Abs}_{control}-{Abs}_{sample}}{{Abs}_{control}}\times 100$$

The ABTS●^+^ radical was generated by mixing 7 mM ABTS with 2.45 mM potassium persulfate (K_2_S_2_O_8_) in a 1:1 ratio, followed by incubation in the dark at room temperature for 16 h. The resulting ABTS solution was diluted with methanol until its absorbance reached 0.7 at 734 nm. Gallic acid was used as the reference standard, and a calibration curve was prepared based on the inhibition (%) values at different concentrations. Ulvan-based samples were tested at a concentration of 0.5 mg/mL. For the assay, 10 μL of either the sample or standard was added in triplicate to 96-well plates, followed by 200 μL of ABTS solution. The mixture was incubated in the dark at room temperature for 30 min, and absorbance was measured at 734 nm using a UV-VIS spectrophotometer. The percentage of ABTS●^+^ radical scavenging activity was calculated using the same inhibition formula described above [32,47].

#### 4.4.6. Cell Viability Assay and Scratch Wound Healing Assay

Cytotoxicity analysis was performed using the Resazurin assay. Cells are seeded into 96-well plates at a density of 1 × 10^5^ cells per well. After overnight incubation at 37 °C in a humidified incubator with 5% CO_2_, cells were treated with Ulvan, Formulation YY-1, and boric acid, at concentrations ranging from 5 to 50 ng/mL. Following 24 h of incubation under the same conditions (37 °C, 5% CO_2_), Resazurin solution (Sigma-Aldrich, R7017) is prepared at a final concentration of 0.02% in PBS and added to each well. After incubation with Resazurin, fluorescence intensity was measured using a microplate reader (Thermo Scientific, Varioskan® Flash, Waltham, MA, USA) set to excitation at 570 nm and emission at 590 nm.

The stimulatory effects of the molecules introduced on the migration capacity of 3T3 mouse fibroblast cells are determined by scratch wound assay. Cells are seeded into 24-well plates at a density of 200,000 cells/well. After incubation in DMEM supplemented with 10% fetal bovine serum (FBS), 100 IU/mL penicillin and 100 μg/mL streptomycin, at 37 °C in a humidified atmosphere containing 5% CO_2_ for 24 h, the culture medium was removed and the cell layer is gently scratched by using a sterile 200 µL pipette tip. Subsequently, 500 µL of culture medium containing Ulvan, Formulation YY-1, boric acid at a concentration of 10 µg/mL is added to each well. Wound closure was monitored by capturing images at 0, 4, 6, and 24 h using Olympus CKX41 inverted microscope. Wound closure images were analyzed using ImageJ 1.54f (Bethesda, MD, USA). The wound area was quantified using the ‘Measure’ tool after scale calibration. Migration velocity was calculated as the change in wound area divided by elapsed time (µm·h^−1^). Data are presented as mean ± SD (*n* = 3) and analyzed statistically using one-way ANOVA (*p* < 0.05).

### 4.5. Rheological Measurements

Rheological measurements were conducted using an Anton Paar MCR 301 rheometer (Berlin, Germany) equipped with a cone–plate geometry (cone angle: 1°, diameter: 50 mm). Prior to testing, each sample was rested on the plate for 5 min to eliminate residual shear history. All measurements were performed under precise temperature control (±0.05 °C). Two types of tests were carried out: (i) Flow behavior: The apparent viscosity (η) was determined as a function of shear rate (γ = 0.1–1000 s^−1^) under rotational controlled shear rate (CSR) conditions to assess whether the samples exhibited Newtonian or non-Newtonian behavior. (ii) Viscoelastic behavior: The storage modulus (G′) and loss modulus (G″) were measured by oscillatory frequency sweep tests (ω = 0.1–100 s^−1^). The linear viscoelastic region (LVE) was first determined from amplitude sweep experiments performed at a fixed angular frequency (ω = 10 s^−1^) with strain amplitudes ranging from 0.01% to 500%.

### 4.6. Statistical Analysis

All experiments were carried out in triplicate (*n* = 3). Results are expressed as mean ± standard deviation (SD). Statistical analyses and graph plotting were performed using GraphPad Prism 10.0 software (GraphPad Software, San Diego, CA, USA) for cytotoxicity studies and for the other data OriginPro 2023 (OriginLab Corporation, Northampton, MA, USA) was used. One-way analysis of variance (ANOVA) followed by Tukey’s post hoc test was used to determine significant differences between groups. Statistical significance was accepted at *p* < 0.05.

## Figures and Tables

**Table 1:** Comparative properties of UP6 and UP7 for the selection of an optimal matrix for YY formulation.

Property	UP6 (AA/PVA/Ulvan 35:30:35)	UP7 (PVA/Ulvan 80:20)
Swelling in PBS (max, %; mean ± SD)	1700% (±5)	1400% (±5)
ABTS inhibition @0.5 mg/mL (%)	20.7 ± 1.2%	40.3 ± 1.5%
DPPH inhibition @0.5 mg/mL (%)	18.2 ± 1.1%	42.1 ± 1.8%
DSC main transitions (°C)	41; 223	50; 123; 226
SEM (qualitative pore/connectivity)	Porous, Ulvan particles distributed partly homogeneously within the matrix	Less porous, Ulvan particles distributed homogeneously within the hydrogel matrix
Practical processing notes	The final formulation is not homogenous being present gel particles	Forms homogeneous a cream-textured material when mixed with the other actives

**Table 2:** Summarized data for the migration rate of the cells after various time intervals for the tested samples. The presented data are the average of three parallel experiments, mean± 0.5 (*n* = 3).

RATE OF MIGRATION (µm/h)			
Group	4H	6H	24H
Ulvan (10 µg/mL)	22.11 ± 1.5	28.79 ± 4.32	27.25 ± 4.09
YY-1 (10 µg/mL)	45.18 ± 6.78	39.92 ± 5.99	27.19 ± 4.08
Boric Acid (10 µg/mL)	20.20 ± 3.03	26.20 ± 3.93	23.86 ± 3.58
Control	4.59 ± 0.69	13.94 ± 2.09	24.80 ± 3.72

**Table 3:** The prepared hydrogels and formulation.

Sample Code	Composition (wt%)	Solid Content (wt%)	Preparation Method
UP1	HA/PVA/Ulvan (50:30:20)	4	Freeze–thaw, 2 cycles, 20 h at −20 °C and thawing for 4 h at RT followed by freeze-drying (lyophilization) to obtain white-like sponges to be tested for further characterization
UP2	CS/PVA/Ulvan (50:30:20)	4	
UP3	HA/PVA/Ulvan (35:30:35)	4	
UP4	CS/PVA/Ulvan (35:30:35)	4	
UP5	AA/PVA/Ulvan (50:30:20)	4	
UP6	AA/PVA/Ulvan (35:30:35)	4	
UP7	PVA/Ulvan (80:20)	4	
Semi-solid YY	Semi-solid formulation prepared by mixing Ulvan-based biopolymer (UP 7) with other ingredients such as UP7 4.5%; Boric acid 2.3%; ZnO 3.4%; CMC 1.2%; FE 0.5%; B 0.5%; and water 88 *w*/*w*.	10	The formulation was prepared by mechanical stirring by using an overhead mechanical stirrer with a speed between 200 and 800 rpm. After homogenizing and obtaining cream-like material for analysis purposes some part of the sample was lyophilized.

CMC—carboxymethyl cellulose; FE—phenoxyethanol; B—*Boswellia serrata* (frankincense); RT—room temperature.

## Data Availability

Details on data and results are available upon request from the corresponding author.
